# How stress influences short video addiction in China: an extended compensatory internet use model

**DOI:** 10.3389/fpsyg.2024.1470111

**Published:** 2024-11-08

**Authors:** Huiyuan Hu, Meilin Huang

**Affiliations:** Taofen School of Journalism and Communication, East China University of Political Science and Law, Shanghai, China

**Keywords:** short video addiction, compensatory internet use (CIU) theory, PLS-SEM, immersion, motives, attitude, perceived stress

## Abstract

**Introduction:**

The rise of short video applications has become a defining feature of modern digital media consumption, drawing increasing attention from researchers due to issues related to short video addiction. While earlier studies have examined the perceived stress as a cause of short video addiction, there is limited understanding of the potential mechanisms underlying the relationship between these two variables. Building on compensatory Internet use (CIU) theory, this study introduces an extended model (E-CIU) to explore how stress, compensatory motivations (i.e., social interaction and relaxing entertainment), and affective responses (i.e., immersion and attitude) relate to short video addiction. This study also examines differences between the age groups.

**Methods:**

Data from 319 Chinese short video users were tested applying partial least squares structural equation modeling (PLS-SEM) and PLS-SEM multigroup analysis.

**Results:**

Findings indicate that stress, immersion, and attitude each contribute positively to short video addiction. Stress is linked to both social interaction and relaxing entertainment. While both factors positively affect attitude toward short videos, only relaxing entertainment enhances immersion. Results confirmed the perceived stress indirectly influences short video addiction through a serial mediating pathway comprising motivations and affective responses. Moreover, the study shows that perceived stress influences social interaction, relaxing entertainment influences attitude and immersion, and social interaction influences immersion across all age groups. The study further identified variations in how different groups experience the relationship between stress and addiction, stress and relaxation, attitude and addiction, and immersion and addiction.

**Discussion:**

Consequently, this study enriches the understanding of the E-CIU as a new theoretical model of short video addiction. These insights offer practical recommendations for short video applications to address user engagement and addiction more effectively.

## 1 Introduction

In recent years, individuals have increasingly faced stress from various aspects of modern life, including personal, professional, academic, familial, and social domains. Stress is generally understood as a state where external demands exceed internal resources, triggering the neuroendocrine stress response, which can lead to anxiety, depression, and other negative emotional states (Lazarus, 1993). To manage these pressures, individuals often turn to different coping strategies. Online media has become a popular and effective method for stress relief, encompassing video games, social network sites, and general internet (Cannito et al., 2022; Huang et al., 2021; Qin et al., 2022; Velezmoro et al., 2010; Zhang et al., 2019). Short videos, with their characteristics of mobility, interactivity, easy access, and diverse content, greatly satisfy the multiple needs of Chinese netizens and rapidly develop into a “new favorite” of online media for daily usage (Jiang and Yoo, 2024). The 53rd China Internet Network Information Center Statistical Report on Internet Development reveals that as of December 2023, there are 1, 053 billion short video users in China, constituting 96.4% of the total number of internet users (China Internet Network Information Center, 2023). Moreover, the China Network Audio-Visual Development Research Report discloses that people spend approximately 151 min daily watching short videos (National Radio and Television Administration, 2024). In light of such excessive use, issues of short video addiction have surfaced (Zhang et al., 2019). Short video addiction can be described as “a condition in which individuals invest considerable time using short video apps, despite encountering adverse outcomes” (Qu et al., 2024, p.1), which precipitates a plethora of negative consequences for users, including increased depressive symptoms, reduced subjective wellbeing, and deteriorated parent–child relationships (Mu et al., 2022; Jiang and Yoo, 2024; Qu et al., 2024).

Existing research indicates a significant positive correlation between stress and short video addiction (Mu et al., 2022; Huang et al., 2021; Liu et al., 2021; Zhang et al., 2019). This addiction is seen both as a compulsive behavior resulting from loss of self-control (Chak and Leung, 2004; Kim et al., 2017) and a conscious self-compensation strategy to manage negative emotions (Kardefelt-Winther, 2014a; Shen and Williams, 2011). The former provides an approach for researching the relationship between self-control and addiction, while the latter allows an empirical examination of how negative emotion influences addictive behavior within a compensatory approach. This paper would prefer the latter approach. Meanwhile, researchers suggest that the stress-short video addiction link can be explained by metacognition and self-compensatory motives (i.e., escape and coping motives) (Huang et al., 2021; Liu et al., 2021; Sun et al., 2024). However, prior studies have not taken into account the associations of affective responses and their mediating roles between stress and short video addiction. The answer to this key question would help us further understand the mechanism underlying the influence of stress on short video addiction. Furthermore, individuals across different age groups exhibit varying cognitions and responses to stress and short video addiction. On the one hand, these differences arise from the distinct pressures faced by each age group, as well as their varying levels of pressure perception (Birditt et al., 2021). On the other hand, speculation of “addiction varies with age” by Lu et al. (2022) suggesting that the degree of addiction also differs among individuals at various life stages. Previous studies neglected the potential influence of age as a moderating role. Specifically, we would explore whether there were differences in the relationship between stress and short video addiction in age groups (i.e., adolescents, emerging adulthood, adulthood).

The compensatory internet use (CIU) theory provides a suitable framework for addressing the mechanisms between stress and addiction as a core research question. This model has gained wide acceptance in the addiction field (e.g., Gong et al., 2021; Stanković et al., 2021; Kardefelt-Winther, 2014a, b). Base on the conventional CIU model, this study proposes an extended compensatory internet use model (E-CIU), introducing the key variables of affective responses (i.e., attitude and immersion) and further exploring their relationship with perceived stress, compensatory motivations, and short video addictive behavior. More specifically, the current research aims to address two main questions within the E-CIU framework: (1) examining the effects of stress, compensatory motivations and affective responses on short video addiction; (2) exploring how the age groups moderate the relationship between these variables above.

## 2 Theoretical background

### 2.1 The compensatory internet use theory (CIU)

Compensatory internet use theory focuses on explaining why individuals become addicted to the internet (Kardefelt-Winther, 2014a). The theory suggests that individuals may compensate in the online world for unmet needs in real life. When people’s negative emotions cannot be effectively vented in real life, individuals may use the internet as an escape or coping strategy to reduce the impact of negative emotions, which may lead to internet addiction in the long run (Kardefelt-Winther, 2014a). According to the compensatory internet use theory, the degree of an individual’s short video addiction is influenced by negative emotional states, particularly stress perception (Liu et al., 2021). Individuals could experience various forms of stress in daily life, including academic pressure, employment challenges, work-related stress, and familial obligations. To cope with these pressures, individuals tend to relieve themselves by using short video applications, which leads to excessive usage of short videos or short video addiction.

The compensatory internet use theory also holds the importance of self-compensatory motivations, and highlights the variations in different contexts. For instance, the compensatory purposes of video games include achievement, social interaction, and immersion (Kardefelt-Winther, 2014b), while the compensatory purposes of social network service include information search and entertainment (Luchman et al., 2014), as well as maladaptive mood regulation (LaRose et al., 2003; Caplan, 2005). Short video applications such as TikTok, Kwai, and Bilibili allow users to upload short videos of 10 of seconds or minutes from their daily lives, such as cooking, singing, dancing, traveling, and health, for other users to watch (Zhang et al., 2019). These UGC contents are interesting and have addictive hedonic value (Tian et al., 2023; Cui et al., 2022; Zhang et al., 2019). These applications also provide functions such as “like,” “comment,” or “forward” to meet users’ social needs (Da-yong and Zhan, 2022). Therefore, this study classifies the compensatory motives of users who choose to use short video applications as relaxing entertainment and social interaction, and takes them as mediating factors to analyze the mechanisms between stress and short video addiction.

### 2.2 An extended model of CIU theory (E-CIU)

The compensatory internet use theory reveals the mediating role of compensatory motivations (e.g., social interaction and relaxing entertainment) in the relationship between negative emotions (i.e., stress) and internet addiction behaviors (Kardefelt-Winther, 2014a, b), but overlooks the possibility of other factors related to affective responses. Prior studies have found that motives positively predict affective responses (Karagiannidis et al., 2015), suggesting that once users perceive the favorable value of using mobile service, they respond with certain positive experiences. For instance, Pang (2021) demonstrated that individuals’ positive attitude and gratification toward social media usage was impacted by hedonic and utilitarian values. Simultaneously, affective responses have a significant positive impact on short video addiction, such as curiosity, and affinity (Dong et al., 2024). Therefore, we contend that it is necessary to integrate other affective factors into the original CIU model and construct an extended CIU model to improve the explanatory power of the short video addiction behavior.

Attitude is an important factor that effectively predicts online addictive behavior (Can and Kaya, 2016; Jeong and Kim, 2011; Tsai and Lin, 2001). Previous studies indicated that attitude should be conceptualized as a tripartite structure, primarily consisting of affective (e.g., emotion or feeling), cognitive (e.g., beliefs, judgments, or thoughts), and behavioral information domains (Edwards, 1990; Bizer and Krosnick, 2001). Nevertheless, scholars’ definitions of attitude show their recognition of the affective component. For example, a handful of extant studies suggested that attitude refers to the positive and negative views held by an individual toward a specific object, such as an action, event, situation, issue, or people (Howarth, 2006; Marcinkowski and Reid, 2019). Ozel et al. (2013, p. 13) also indicated that attitude reflects “a general liking or disliking, or more specific affective reactions toward the object.” Similarly, Petty and Briñol (2015, p. 2) have posited that “attitudes are not only based on thoughts and beliefs but also feelings and emotions.” In addition, existing studies have also regarded attitude as a factor of affective response when constructing theoretical models (Henter, 2014; Pang, 2021; Zhang, 2013). Therefore, attitude is one of the affective factors considered in this study.

Generally, immersion has been recognized as one of the most significant conceptions for understanding users’ affective experience in the field of media effect (Cheng and Tsai, 2020; Lin et al., 2020). Immersion refers to “a state of deep mental involvement in which their cognitive processes (with or without sensory stimulation) cause a shift in their attentional state such that one may experience dissociation from the awareness of the physical world” (Agrawal et al., 2020, p. 407). Jennett et al. (2008) analyzed the differences between immersion and relevant concepts such as flow, cognitive absorption, and presence in detail. They argued that the key to the construct of immersion lay in emphasizing the loss of awareness of time and the real world, involvement, and focused attention. Building upon this foundation, this study believes that when individuals watch short videos they like or prefer, they have a good viewing experience and a sense of being “lost to the world” (Seah and Cairns, 2008). At present, most studies on immersion and addictive behaviors focus on video games (Lee et al., 2021; Seah and Cairns, 2008), virtual reality services (Saneinia et al., 2022), and gambling (Rémond and Romo, 2019), the role of immersion in short video addiction also needs to be empirically tested. Thus, this study proposes attitude and immersion as the affective factors, we aim to empirically investigate how stress perception affects addictive outcomes through motivational and affective paths in the context of short video applications.

### 2.3 Hypothesis development

#### 2.3.1 Perceived stress and short video addiction

Perceived stress, conceptualized by Cohen et al. (1983) as “the degree to which individuals appraise situations in their lives as stressful,” has been shown to instigate a cascade of responses at the psychological, behavioral, and physiological levels (Schiffrin and Nelson, 2008). This multifaceted impact extends to individuals’ susceptibility to addiction, particularly in relation to the emerging phenomenon of short video addiction. Recent research by Liu et al. (2021) elucidates that perceived stress serves as a pivotal determinant directly influencing the addictive tendencies toward short video applications among Chinese users. Moreover, the literature highlights that the alleviation of stress and facilitation of relaxation serve as central incentives driving the consumption of mobile videos (McNally and Harrington, 2017). Given the established link between perceived stress and short video addiction, we hypothesize that:

*H1*: Perceived stress is positively associated with short video addiction.

#### 2.3.2 Perceived stress, relaxing entertainment, and social interaction

Stress, as a negative emotional experience, drives individuals to seek relief. Short video applications provide a notable coping strategy for stress reduction (Sun et al., 2024). According to the compensatory internet use theory, internet addiction can arise from individuals seeking gratifications online to alleviate negative emotions. This paper will examine two key aspects of gratifications-seeking in relation to short videos: relaxing entertainment and social interaction. These elements align with the use and satisfaction theory. Meanwhile, Vaterlaus and Winter (2021) reviewed existing literature on short video user motivations and found significant overlap in the focus on relaxation entertainment and social interaction.

Previous studies demonstrate the influence of perceived stress on motives for media consumption. Pavić and Rijavec (2013) highlight the significant effect of perceived stress on instrumental motives and ritual motives toward a television viewing environment. A study by Sun et al. (2024) based on empirical data found that stress can stimulate people’s escape motive for using short videos. This view indicates that if individuals have a higher level of experienced stress, they are more likely to generate the motivation to watch short videos to achieve escape. This study aimed to test whether a positive association between stress and relaxing entertainment motive and social interaction motive. Under significant pressure (e.g., work stress, academic stress, etc.), people often turn to short videos for relief, seeking both entertainment and social interaction. Consequently, we formulate hypotheses as following:

*H2*: Perceived stress is positively associated with relaxing entertainment.

*H3*: Perceived stress is positively associated with social interaction.

#### 2.3.3 Relaxing entertainment, social interaction and attitude

Previous studies found that social interaction and relaxing entertainment are the two most important motivations for using short video applications (Zhang et al., 2023; Shi et al., 2024; Deng et al., 2023). On the one hand, short videos have the function of relaxation and entertainment. Chen and Lin (2018) noted that the purpose of relaxing entertainment is to make users feel happy by providing a temporary escape from reality, allowing them to relieve stress by forgetting their worries. Dong and Xie (2024) report that users regard short video applications as relaxing entertainment to relieve stress. On the other hand, Dholakia et al. (2004, p. 244) defined social interaction as “the social benefits derived from establishing and maintaining contact with other people such as social support, friendship, and intimacy.” Vaterlaus and Winter (2021, p. 9) noted that TikTok, one typical short video application, by nature, is “a relational activity or a way to form new relationship.” Thus, users prefer to maintain online interpersonal relationships on the short video applications by adding friends, chatting with friends, sharing, liking, commenting, and other interactive activities. Past studies have found that both relaxing entertainment and social interaction positively impact attitude. For example, Curras-Perez et al. (2014) concluded that the motivations of interacting with friends, meeting new users, finding enjoyment, entertainment, and escapism are strong predictors of the attitude toward social network site usage. Therefore, this study proposes the following two hypotheses:

*H4*: Relaxing entertainment is positively associated with attitude.

*H5*: Social interaction is positively associated with attitude.

#### 2.3.4 Relaxing entertainment, social interaction and immersion

Drawing on flow theory (Csikszentmihalyi, 1990), we believe that users are eager to seek a high level of interactive experience through short videos and positively influence their immersion. Empirical studies have shown that motivation is considered to be a proximal determinant of immersion, and the underlying mechanism of this effect is users’ expectation of positive outcomes from internet use (Liu and Chang, 2016; Miranda et al., 2023). The primary goal of short video usage is to create entertainment and interaction through intrinsic motivation, which is closely related to flow and immersion (Yan et al., 2023). Tian et al. (2023) found that social interaction and entertainment are also positively correlated with immersion. In a study conducted by Lv et al. (2022), showed that entertainment and social interaction play significant roles in user immersive experience. In addition, Miranda et al. (2023) found in a study of short video addictive behaviors that avoidance motivation increased individual immersion. Therefore, we believe that entertainment and social interaction positively influence the formation of immersion because they enrich the viewing experience and produce high levels of concentration and cognitive absorption. Thus, we propose the following hypotheses:

*H6*: Relaxing entertainment is positively associated with immersion.

*H7*: Social interaction is positively associated with immersion.

#### 2.3.5 Attitude and short video addiction

Attitude toward behavior delineates a user’s endorsement of spending more time-consuming short videos. When users perceive short videos as delivering values such as entertainment and relaxation, they develop a favorable attitude toward short videos, and then their behavior becomes proactive. Precious studies, such as Lai et al. (2016), explored the impact of favorable attitude on users’ addiction to online gaming applications. Hence, we embrace Lai et al.’s (2016) viewpoint in the present study to understand how attitude influences individuals’ short video addictive behavior. Hence, we postulate the following research hypothesis:

*H8*: Attitude is positively associated with short video addiction.

#### 2.3.6 Immersion and short video addiction

Lee and Li (2023) suggested that immersion represents a sense of cognitive absorption, concentration, and forgetting about physical reality. In an empirical study, Lehenbauer-Baum et al. (2015) pointed out that immersion availability could enhance individuals’ MMORPG addiction. In the present study, immersion refers to users becoming immersed in a video-centric world, such as diverse content, and interactive experiences like liking. Besides, with its streamlined algorithmic recommendations, short video applications can rapidly discern, comprehend, and assimilate user behavior. Sustained delivery of tailored content enhances user engagement with the viewing experience and may contribute to a propensity for excessive short video consumption. Ye et al. (2023) argued that the deeply immersive nature of short videos may lead to heightened difficulty for users in disengaging. Moreover, previous research demonstrated a positive correlation between immersion and short video addiction (Nong et al., 2023; Yang et al., 2021). Thus, this study proposes the following hypothesis:

*H9*: Immersion is positively associated with short video addiction.

#### 2.3.7 Multigroup difference

Age has been widely used as an important moderating variable in internet addiction studies (Chatterjee, 2021; Ioannidis et al., 2018). Previous studies have shown that users of different ages show different levels of addiction. In the study of Heo et al. (2014), Korean high school students have a low addictive internet use score, which is related to high academic performance pressure. Devine et al. (2022) found that younger adults tended to have greater levels of internet addiction than older adults. Previous studies highlight the differences among different age groups. In addition, it has been found that age regulates the relationship between antecedents and addiction. Chatterjee (2021) found that, compared with young adults (18–35 years), middle-aged adults (36–55 years) had a stronger predictive effect on internet addiction, such as loneliness and anxiety. In our study, according to the age classification recommended by the National Bureau of Statistics of China, we divided young short video users into three categories and named them: adolescence (14–20 years old), emerging adults (21–25 years old), and adulthood (>25 years old), and the number of young short video users in these three categories is relatively high in China. In this article, we will discuss how individuals in these three age groups influence the associations of the six variables in the proposed model. Therefore, this paper proposes the following research questions:

RQ1: Does Age moderate the relationships among the stress, relaxing entertainment, social interaction, attitude, immersion and short video addiction

**Figure 1:**
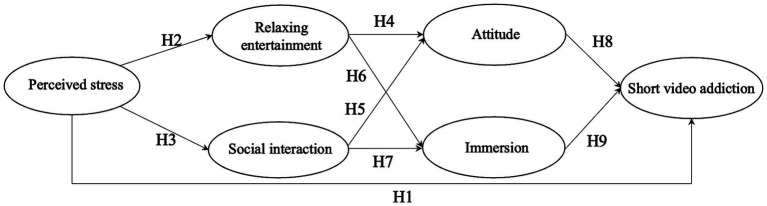
Research model.

The conceptual model diagram is illustrated in Figure 1.

## 3 Materials and methods

### 3.1 Measures

Following rigorous translation and back-translation, all English measurement items involved in this research were translated into Chinese by the authors. The questionnaire used in this survey mainly consists of two parts and two additional screening question. First, participants need to answer one screening questions, which asks whether they have the habit of watching short videos in the past 6 months. Participants who choose “Yes” are invited to fill in the remaining survey questions. In contrast, those who choose “No” are denied to participate. The first part measures the model variables, and the second part is descriptive statistical analysis, including variables such as gender, age, monthly income, education level, and average daily usage time (Table 1). Additionally, an attention-check question was inserted in the middle of a questionnaire that helps to screen out invalid questionnaires. In total, 30 questions of the questionnaire were encompassed in this paper.

**Table 1:** Sample profile (*N* = 319).

Variables	Distribution	Frequency	Percent (%)
Gender	Male	148	46.4%
	Female	171	53.6%
Age	14–20	70	21.9%
	21–25	178	55.8%
	26–30	44	13.8%
	31–35	11	3.4%
	36 and more	16	5.0%
Education level	Middle school or lower	33	10.3%
	High school	66	20.7%
	Junior college	23	7.2%
	College	128	40.1%
	Master’s or higher	69	21.6%
Average daily usage time	<30 min	84	26.3%
	30-60 min	87	27.3%
	1-2 h	83	26.0%
	2-3 h	39	12.2%
	>3 h	26	8.2%
Monthly income (CNY)	3,000 or less	192	60.2%
	3,001–5,000	55	17.2%
	5,001–8,000	40	12.5%
	8,001–10,000	11	3.4%
	10,001or more	21	6.6%

To further enhance the validity of the questionnaire and ensure that each item could represent a specific construct, we invited four experienced researchers and four master students in the field to modify it and obtained their approval. Subsequently, we conducted a pre-survey with 82 participants to evaluate whether the reliability and validity of the six scales met the thresholds proposed by previous studies. We undertook additional modifications to the questionnaire scale in response to the confusion and feedback provided by participants following their completion of the survey. The results of the preliminary survey are satisfactory, and then we can proceed to distributing the formal version’s questionnaire. In the first part of the questionnaire, the measurement items were evaluated using a 5-point Likert scale, ranging from “1 = strongly disagree” to “5 = strongly agree.” Specifically, attitude was measured in three items using Ajzen’s (2002) and Eagly and Chaiken’s (1993) scale. Following Khan (2017), relaxing entertainment was measured by using two items. The measurement of immersion was adopted from Hamari et al. (2016). Social interaction was measured in three items using Zadeh et al. (2023) scale. The measurement of perceived stress was based on Cohen et al.’s (1995) scale. Moreover, short video addiction was adopted from Chen et al. (2003). All measurement items are included in the Table 2.

**Table 2:** Instrument of the variables.

Variables (sources)	Items
Perceived stress (Cohen et al., 1995)	I am unable to cope with life’s challenges and feel overwhelmed or stressed.
	I cannot control important matters and worries in life and feel depressed.
	I lack confidence in dealing with significant changes happening in my life.
	I feel incapable of overcoming difficulties and making things progress smoothly.
Relaxing entertainment (Khan, 2017)	To relax.
	To pass the time when bored.
Social interaction (Zhang et al., 2023)	To meet new people
	To interact with people with the same interests
	To spend time with people I care about
Attitude (Ajzen, 2002; Eagly and Chaiken, 1993)	I enjoy watching short videos for a long time.
	I think watching short videos for a long time is normal behavior.
	I think watching short videos for a long time is beneficial.
Immersion (Hamari et al., 2016)	I find it easy to concentrate and immerse myself in watching short videos, often losing track of time.
	I often immerse myself in short videos recommended by short video apps.
	I often immerse myself in short videos I am interested in.
Short video addiction (Chen et al., 2003)	I watch short videos whenever I have free time.
	I open the short video application frequently.
	I feel that life without the short videos would be boring, empty and joyless.
	I feel that I watch short videos longer than before.
	I neglect the interaction with my family, friends, classmates and colleagues to spend more time watching short videos.
	On more than one occasion, I have delayed things that need to be completed on time because of watching short videos.
	I try to cut down the amount of time I spend online, but failed.

### 3.2 Sampling and data collection

The formal survey was conducted from June 3rd to July 5th, 2021, utilizing the Sojump^1^ online platform, which boasts a user base of over 10 million. This study obtained approval from the institutional review board (IRB) of the author’s affiliated institution. A total of 356 questionnaires were distributed, and after excluding invalid responses such as those from non-short video users and those with identical consecutive answers exceeding 10 instances, a total of 319 valid questionnaires were collected, resulting in a response rate of 89.60%. Table 1 presents the sample characteristics. Among all 319 participants, 46.4% (*n* = 148) were males and 53.6% (*n* = 171) were females. 68.9% of them had a bachelor’s degree or higher and 46.4% of the participants engaged in daily consumption of short videos for over 1 h.

### 3.3 Statistical analysis

SPSS 24.0 and SmartPLS 4.0 were used to analyze the data. This study used SPSS 24.0 to conduct a descriptive analysis of respondent demographic characteristics analysis. Then, a two-step approach was used for PLS-SEM analysis. In the initial step, the reliability, content validity, and discriminant validity of the measurement model were evaluated. In the second step, path analysis and multigroup analysis was applied to test the proposed research hypotheses in this study.

## 4 Results

### 4.1 Measurement model

The validity and reliability of the measurement were assessed through confirmatory factor analysis using SmartPLS 4.0. As shown in Table 3, the Cronbach’s alpha values exceeded a marginal value of >0.7, and Composite Reliability (CR) for each construct was higher than 0.7, which is a widely accepted threshold in research, indicating good internal consistency reliability (Hair et al., 2017). The Average Variance Extracted (AVE) values for all constructs ranged from 0.685 to 0.880, surpassing the threshold of >0.5, and all Outer Loadings (OL) were greater than the suggested value of 0.7, leading to the conclusion that convergent validity is not a concern (Bagozzi and Yi, 1988). The findings, as presented in Table 4, confirmed the discriminant validity by showing that the square root of the AVE (highlighted in bold and displayed on the diagonal figure) surpassed its correlation coefficients with other factors. Hence, the present study has favorable discriminant validity (Fornell and Larcker, 1981).

**Table 3:** Instrument of the variables of the standardized factor loading value and scale reliability.

Indicators	Abb.	Items	Factor loading	Cronbach’s α	*CR*	*AVE*
Attitude	ATT	ATT1	0.847	0.803	0.810	0.716
		ATT2	0.855			
		ATT3	0.837			
Relaxing entertainment	RET	RET1	0.923	0.783	0.799	0.820
		RET2	0.889			
Immersion	IMM	IMM1	0.828	0.778	0.810	0.689
		IMM2	0.785			
		IMM3	0.875			
Social interaction	INT	INT1	0.896	0.783	0.799	0.820
		INT2	0.869			
		INT3	0.900			
Perceived stress	PSS	PS1	0.920	0.954	0.956	0.880
		PS2	0.953			
		PS3	0.946			
		PS4	0.933			
Short video addiction	SVA	SVA1	0.887	0.923	0.934	0.685
		SVA2	0.893			
		SVA3	0.866			
		SVA4	0.842			
		SVA5	0.751			
		SVA6	0.736			
		SVA7	0.806			

CR represents “Composite Reliability,” AVE represents “Average Variance Extracted”.

**Table 4:** Fornell-Larcker criterion.

	Mean	SD	1	2	3	4	5	6
1. ATT	2.82	0.997	**0.846**					
2. PSS	3.08	1.060	0.481	**0.938**				
3. SVA	2.86	0.950	0.575	0.680	**0.828**			
4. RET	3.79	0.855	0.496	0.497	0.464	**0.906**		
5. IMM	3.68	0.843	0.374	0.524	0.554	0.617	**0.830**	
6. INT	2.52	1.051	0.596	0.489	0.540	0.330	0.269	**0.888**

ATT, Attitude; RET, Relaxing entertainment; IMM, Immersion; INT, Social interaction; PSS, Perceived stress; SVA, Short video addiction. Numbers in parentheses are square roots of AVE.

In the analysis of the reflective measurement model, we employed the Heterotrait Monotrait Ratio (HTMT) criterion proposed by Henseler et al. (2015) to evaluate discriminant validity among six constructs (see Table 5). The scores of HTMT for all constructs are below the threshold value of 0.85, confirming the discriminant validity of the model.

**Table 5:** Assessment of discriminant validity using the HTMT criterion (HTMT<0.85).

	1	2	3	4	5	6
1. ATT						
2. PSS	0.542					
3. SVA	0.646	0.715				
4. RET	0.611	0.569	0.527			
5. IMM	0.442	0.596	0.613	0.784		
6. INT	0.714	0.538	0.602	0.394	0.308	

ATT, Attitude; RET, Relaxing entertainment; IMM, Immersion; INT, Social interaction; PSS, Perceived stress; SVA, Short video addiction.

### 4.2 Structural model

**Figure 2:**
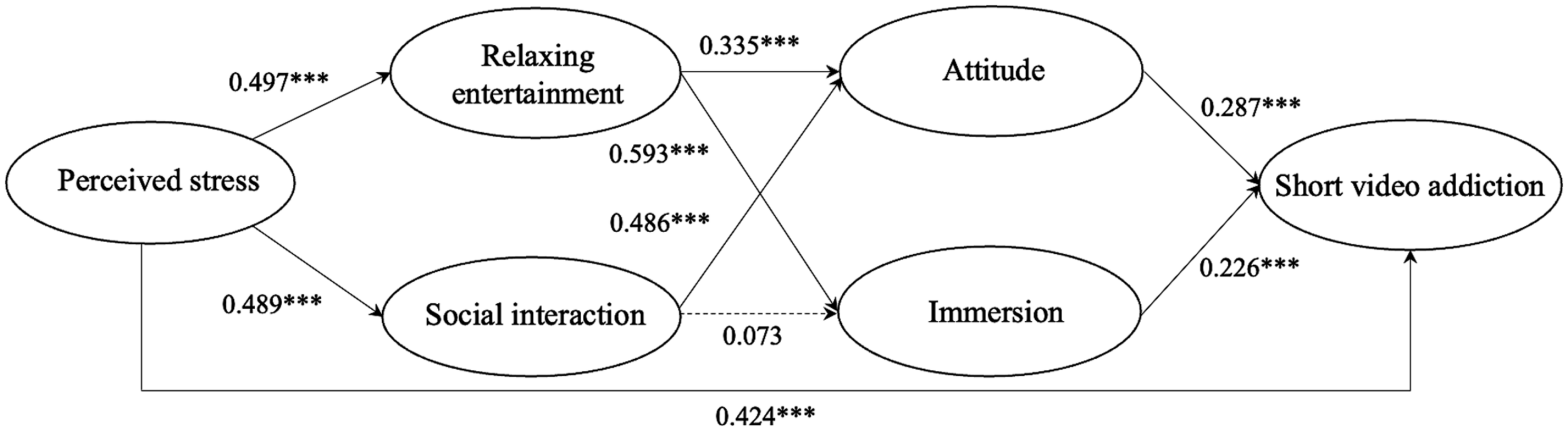
Structural model.

The structural analysis represents the second step of PLS-SEM. To determine the significance of each path coefficient, bootstrapping with 5,000 samples was used. The result of the structural model is depicted in Figure 2, displaying path coefficients, significance levels of paths, *VIF*, *f^2^*, *R^2^* values, and *Q^2^* values. The model explains 24.7% of the variance for relaxing entertainment, 23.9% of the variance for social interaction, 38.5% of the variance for immersion, 45.6% of the variance for attitude, and 57.9% of the variance for short video addiction. This finding showed that the explanatory power of our model is acceptable (Chin, 1998). Furthermore, relaxing entertainment has 0.197, social interaction has 0.186, immersion has 0.254, attitude has 0.317, and short video addiction 0.384 as *Q^2^* values, all *Q^2^* values exceed zero and indicated that the predictive relevance of the structural model was satisfied (Geisser, 1975). In addition, we examined the model fit using the standardized root mean square residual (*SRMR*) measure. Our model had an *SRMR* value of 0.072, which is below the threshold of 0.08 (Henseler et al., 2016), and can be considered an acceptable value for a PLS-SEM-based model.

As shown in Figure 2 and Table 6, the findings from the SEM analysis revealed several key relationships. Perceived stress is positively related to the short video addiction (*β* = 0.424, *p* < 0.001), supporting H1. Perceived stress is positively associated with relaxing entertainment and social interaction (*β* = 0.497, *p* < 0.001; *β* = 0.489, *p* < 0.001). Hence, H2 and H3 were confirmed. Relaxing entertainment displayed significant and positive associations with attitude and immersion (*β* = 0.335, *p* < 0.001; *β* = 0.593, *p* < 0.001). H4 and H6 were supported. Social interaction exhibited noteworthy positive correlations with attitude (*β* = 0.486, *p* < 0.001), but failed to be related to immersion (*β* = 0.073, *p* > 0.05). Therefore, H5 was supported, but H7 was not supported. The effect of attitude on short video addiction is significantly positive (*β* = 0.287, *p* < 0.001). H8 was supported. The results reveal a significant and positive correlation between immersion and short video addiction (*β* = 0.226, *p* < 0.001). Hence, H9 was confirmed.

**Table 6:** Assessment of structural model with bootstrapping procedure.

Path relationship	Std beta	Std error	*t*-value	*p*-value	Confidence interval	VIF	*f^2^*
PSS → SVA	0.424***	0.059	7.223	0.000	[0.324, 0.520]	1.585	0.270
PSS → RET	0.497***	0.048	10.435	0.000	[0.417, 0.574]	1.000	0.328
PSS → INT	0.489***	0.052	9.319	0.000	[0.401, 0.573]	1.000	0.314
RET → ATT	0.335***	0.047	7.154	0.000	[0.257, 0.409]	1.122	0.184
RET → IMM	0.593***	0.046	12.904	0.000	[0.514, 0.666]	1.122	0.510
INT → ATT	0.486***	0.046	10.655	0.000	[0.411, 0.561]	1.122	0.387
INT → IMM	0.073	0.053	1.368	0.086	[−0.016, 0.159]	1.122	0.008
ATT → SVA	0.287***	0.052	5.505	0.000	[0.205, 0.378]	1.337	0.147
IMM → SVA	0.226***	0.047	4.785	0.000	[0.148, 0.304]	1.416	0.085

	Coefficient of determination, *R^2^*	Predictive relevance, *Q^2^*_predict
RET	0.247	0.197
INT	0.239	0.186
IMM	0.385	0.254
ATT	0.456	0.317
SVA	0.579	0.384

ATT, Attitude; RET, Relaxing entertainment; IMM, Immersion; INT, Social interaction; PSS, Perceived stress; SVA, Short video addiction. VIF, Variance Inflation Factor; *f^2^* (Effect Size). **p < 0.010; ***p < 0.001.

### 4.3 Mediating effect test

Following the preceding findings, it is evident that further analysis is warranted to investigate the mediating effects in the relationship between perceived pressure and short video addiction. Mediation analyses using bootstrapping were performed in SmartPLS 4.0 to examine two specific pathways as outlined in Table 7. Given that the positive correlation between social engagement and immersion remains unverified, this study focuses on three specific paths. As indicated by the path analysis results presented in Table 7, perceived pressure influences short video addiction through a serial mediating pathway comprising relaxation entertainment, social interaction, immersion, and attitude.

**Table 7:** Mediation calculation.

	Std beta	Std error	*t*-value	*p*-value	Confidence interval
PSS → RET → IMM → SVA	0.067***	0.016	4.117	0.000	[0.041, 0.094]
PSS → INT → ATT → SVA	0.068***	0.017	3.976	0.000	[0.043, 0.100]
PSS → RET → ATT → SVA	0.048***	0.012	3.997	0.000	[0.030, 0.069]
PSS → INT → IMM → SVA	0.008	0.006	1.236	0.108	[−0.002, 0.019]

ATT, Attitude; RET, Relaxing entertainment; IMM, Immersion; INT, Social interaction; PSS, Perceived stress; SVA, Short video addiction. ***p < 0.001.

### 4.4 Multigroup analysis (MGA)

We use the categorical variable “age group” as a moderator. More specifically, multigroup analysis has sample size requirements: (1) the sample size for each group must be almost equal; (2) no group can use less than 80% of the recommended sample size (Matthews, 2017). Our basic idea is to combine the descriptive statistics of 26–30, 31–35, and 36 and more into one group category “25 and more,” which further divide the sample into three groups: 14–20 (*n* = 70), 21–25 (*n* = 178), and 25 and more (*n* = 71). However, we found that the sample size in each group was not equal, which would lead to weakened statistical power and the underestimation of the moderating effect (Hair et al., 2017; Aguinis et al., 2017). According to the recommendations of Aguinis et al. (2017) and Matthews (2017), we chose almost equal sample sizes. The main method was to use the random sampling method in the Excel software from the 21–25 (*n* = 178) group and select 71 participants.

Following the procedure suggested by Cheah et al. (2020), we first used the measurement invariance of composite models (MICOM) to assess measurement equivalence, which can be effectively explained if the research data standards are acceptable. More precisely, this study aimed to ascertain whether construct measurements are understood similarly across the three age groups. MICOM consists of three steps, the first step is the configurational invariance assessment, the second is the establishment of compositional invariance assessment and the third is the assessment of equal means and variances. The results as shown in Table 8, the MGA’s group-specific differences in the PLS-SEM findings are feasible to compare and interpret (Henseler et al., 2016) and are acceptable to perform path analysis among three groups.

**Table 8:** Assessment of measurement invariance.

Comparison	Constructs	Configurational invariance (Step 1)	Compositional invariance (Step 2)		Partial measurement invariance	Equal mean assessment (Step 3a)		Equal mean assessment (Step 3b)		Full measurement invariance
			Original Correlation	5.0%		Original Differences	Confidence Interval	Original Differences	Confidence Interval	
Group 14–20 Vs. Group 21–25	ATT	Yes	0.999	0.985	Yes	0.119	[−0.323, 0.329]	−0.082	[−0.539, 0.474]	Yes/Yes
	PSS	Yes	1.000	1.000	Yes	0.021	[−0.348, 0.349]	−0.512	[−0.562, 0.446]	Yes/Yes
	SVA	Yes	0.999	0.998	Yes	0.264	[−0.330, 0.319]	−0.172	[−0.492, 0.471]	Yes/Yes
	RET	Yes	0.998	0.997	Yes	−0.333	[−0.321, 0.345]	−0.127	[−0.495, 0.476]	No/Yes
	IMM	Yes	0.992	0.991	Yes	−0.63	[−0.340, 0.001]	0.066	[−0.450, 0.402]	No/Yes
	INT	Yes	0.999	0.996	Yes	0.398	[−0.333, 0.017]	−0.166	[−0.536 0.426]	No/Yes
Group 14–20 Vs. Group >25	ATT	Yes	0.999	0.995	Yes	0.005	[−0.314, 0.318]	−0.641	[−0.484, 0.434]	Yes/No
	PSS	Yes	1.000	1.000	Yes	−0.175	[−0.304, 0.346]	−0.583	[−0.482, 0.431]	Yes/No
	SVA	Yes	0.997	0.997	Yes	−0.099	[−0.331, 0.326]	−0.327	[−0.469, 0.463]	Yes/Yes
	RET	Yes	0.999	0.997	Yes	−0.479	[−0.316, 0.328]	−0.210	[−0.477, 0.466]	No/Yes
	IMM	Yes	1.000	0.995	Yes	−0.454	[−0.309, 0.331]	−0.297	[−0.447, 0.408]	No/Yes
	INT	Yes	0.996	0.995	Yes	0.034	[−0.311, 0.328]	−0.387	[−0.478, 0.443]	Yes/Yes
Group 21–25 Vs. Group >25	ATT	Yes	1.000	0.991	Yes	−0.103	[−0.323, 0.310]	−0.561	[−0.447, 0.463]	Yes/No
	PSS	Yes	1.000	1.000	Yes	−0.178	[−0.321, 0.318]	−0.073	[−0.390, 0.413]	Yes/Yes
	SVA	Yes	0.999	0.995	Yes	−0.326	[−0.321, 0.297]	−0.152	[−0.431, 0.417]	No/Yes
	RET	Yes	1.000	0.995	Yes	−0.167	[−0.317, 0.336]	−0.074	[−0.474, 0.508]	Yes/Yes
	IMM	Yes	0.993	0.989	Yes	0.153	[−0.315, 0.319]	−0.370	[−0.492, 0.453]	Yes/Yes
	INT	Yes	0.999	0.997	Yes	−0.338	[−0.313, 0.314]	−0.243	[−0.454, 0.453]	No/Yes

AAT, Attitude; RET, Relaxing entertainment; IMM, Immersion; INT, Social interaction; PSS, Perceived stress; SVA, Short video addiction.

From Table 9, our results show a significant difference between Group 14–20, Group 21–25, and Group >25. First, the comparisons between Group 14–20 and Group >25 (|diff| = − 0.574, *p* < 0.01) and Group 21–25 and Group >25 (|diff| = − 0.540, p < 0.001) show a significant relationship between perceived stress and short video addiction. Second, Group >25 significantly differs from Group 14–20 (|diff| = − 0.288, *p* < 0.01) and Group 21–25 and Group >25 (|diff| = − 0.334, *p* < 0.01) for the relationship between perceived stress and relaxing entertainment. Third, the result shows a significant difference between Group 14–20 and Group >25, and Group 21–25 and Group >25 for the relationship between attitude and short video addiction (|diff| = − 0.288, *p* < 0.01, |diff| = − 0.334, *p* < 0.01). Last, the result of the relationship between immersion and short video addiction showed that the Group 14–20 sample differs significantly from the Group >25 sample (|diff| = − 0.334, *p* < 0.05).

**Table 9:** Results of hypothesis testing of multigroup.

	Group 14–20				Group 21–25				Group >25				|diff|		
	Std beta	Std error	*t*-value	*p*-value	Std beta	Std error	*t*-value	*p*-value	Std beta	Std error	*t*-value	*p*-value	Group 14–20 vs. group 21–25	Group 14–20 vs. group >25	Group 21–25 vs. group >25
PSS → SVA	0.214	0.176	1.214	0.112	0.249*	0.118	1.214	0.017	0.788***	0.069	11.499	0.000	−0.035	−0.574**	−0.540***
PSS → RET	0.435***	0.116	3.736	0.000	0.389***	0.105	3.736	0.000	0.723***	0.049	14.64	0.000	0.046	−0.288**	−0.334**
PSS → INT	0.578***	0.138	4.199	0.000	0.441***	0.113	4.199	0.000	0.640***	0.077	8.337	0.000	0.137	−0.062	−0.198
RET → ATT	0.437***	0.102	4.296	0.000	0.341**	0.113	4.296	0.001	0.365***	0.097	3.758	0.000	0.096	0.072	−0.023
RET → IMM	0.638***	0.082	7.748	0.000	0.570**	0.101	7.748	0.000	0.723***	0.079	9.206	0.000	0.068	−0.085	−0.154
INT → ATT	0.462***	0.101	4.559	0.000	0.310**	0.132	4.559	0.009	0.466***	0.086	5.437	0.000	0.152	−0.004	−0.156
INT → IMM	0.164	0.127	1.292	0.098	0.043	0.119	1.292	0.358	0.049	0.087	0.555	0.289	0.121	0.116	−0.005
ATT → SVA	0.299**	0.106	2.828	0.002	0.390***	0.106	2.828	0.000	0.022	0.072	0.301	0.382	−0.091	0.277*	0.368**
IMM → SVA	0.439***	0.126	3.477	0.000	0.269**	0.086	3.477	0.001	0.126*	0.07	1.786	0.037	0.170	0.314*	0.143

ATT, Attitude; RET, Relaxing entertainment; IMM, Immersion; INT, Social interaction; PSS, Perceived stress; SVA, Short video addiction. *p < 0.05, **p < 0.01, ***p < 0.001.

## 5 Discussion

This study employed an extended CIU model to investigate short video addiction in China, highlighting stress as a key factor directly affecting user addiction. By utilizing this model, the research deepens our understanding of the factors driving short video addiction. It integrates self-compensatory motives, immersion, and attitude into the analysis, offering insights specific to the Chinese context. Furthermore, the study presents new opportunities to refine the conventional CIU model by exploring the complex relationships among these predictors.

Firstly, the results revealed a positive correlation between perceived stress and short video addiction, aligning with findings from Liu et al. (2021) and Sun et al. (2024), who reported that higher stress levels are associated with increased addiction to short videos. This relationship can be understood from a psychopathological perspective, stress serves as a mental predisposition and is accompanied by anxiety, which may drive individuals to use short videos more frequently, leading to addiction (Brand et al., 2016). Engaging with preferred short videos can trigger dopamine release, aiding emotional regulation and reducing tension and anxiety (Mouchabac et al., 2021). Consequently, individuals experiencing higher levels of perceived stress and anxiety may seek pleasure through short video consumption, resulting in addictive behaviors.

Secondly, our findings indicate that perceived stress is positively related to two primary motives: relaxing entertainment and social interaction. This finding supports the notion that these motives play a crucial role in compensating for negative emotions, such as stress, among users of short video applications. Specifically, stress has a significant impact on both the relaxing entertainment motive and the social interaction motive. This finding is consistent with previous research (Curras-Perez et al., 2014; Dong and Xie, 2024; Vaterlaus and Winter, 2021), which highlights the importance of relaxation and social interaction as crucial motives when engaging with short video applications. Individuals experiencing higher levels of perceived stress are more likely to watch short videos to fulfill their motives for relaxation and social interaction.

Thirdly, relaxing entertainment has a significant influence on both users’ attitude and immersion, while social interaction only exerts influence on attitude. This finding is consistent with a previous study, confirming a positive association between relaxing entertainment, social interaction, and attitude (Curras-Perez et al., 2014). When users perceive that the short videos consumption satisfies their needs, they are likely to develop a positive attitude toward the usage behavior. Specifically, the desire for relaxation and enjoyment is a crucial driver for using short video applications, which significantly enhances users’ positive attitudes toward the continuous viewing behavior. Additionally, social interaction—such as communication, sharing, and building connections—reflects the social purposes for which users adopt these applications, further influencing their attitudes. Engaging in social interactions on these short video applications can provide a sense of community and belonging, which contributes to a more positive attitude toward the application. For short video application users, relaxing entertainment enhances their immersion, supporting the findings of Tian et al. (2023) and Lv et al. (2022) that the hedonic motive significantly enhances users’ immersive experience.

Fourthly, this study investigates how affective responses, specifically attitude and immersion, predict short video addiction. The findings indicate that both immersion and attitude contribute to increased addiction. Immersion is distinct from related concepts like flow or absorption in this study, and it refers to users losing track of time due to intense focus on short video content, which can lead to addiction and other negative effects (Tian et al., 2023). Many Chinese users report that time seems to fly by while watching short videos, often expressing this with phrases like “5 min on TikTok feels like an hour.” Additionally, the study confirms that attitude significantly correlates with addictive behaviors, which is consistent with prior research in the context of online video games (Jeong and Kim, 2011). Attitude reflects an individual’s general evaluations regarding extended short video watching. Attitudes are critical to decision-making and behavior in that people tend to engage with short video applications (Petty and Briñol, 2015). Thus, users’ positive evaluation of extended short video watching increases the likelihood of addiction.

Moreover, contrary to the expectation, social interaction does not exert influence on immersion. The insignificant linkage here could possibly be attributable to the fact that users who interact with others may be distracted from watching short videos with full attention. As smartphone applications, short video applications function as compelling sources of distraction, effectively capturing user attention through their engaging content (Toh et al., 2023). However, to meet their needs for relaxation, entertainment, and social interaction, users can perform multiple tasks on short video applications, including watching, liking, commenting, joining communities, and even chatting with friends. Research indicates that individuals who multitask less frequently tend to process information sequentially and can fully allocate their attentional resources to a single task (Alloway and Alloway, 2012). Short videos provide a fresh, engaging, and stimulating experience to the audience (Lu et al., 2022; Dong et al., 2024), making them particularly suited for entertainment compared to other video streaming media services. When individuals engage with short videos for entertainment, this viewing activity itself becomes the primary task. Consequently, users are able to filter out extraneous information and derive pleasure from the enjoyment of short videos, thereby achieving a state of immersion. However, when the motivation for watching short videos is rooted in social interaction, the use of these applications often involves multitasking—such as viewing videos while simultaneously monitoring and engaging with the comments and activities of their social network. This multi-task engagement can disperse attentional resources, thereby hindering the attainment of a fully immersive experience.

Fifthly, as an exploratory endeavor, this study examines how users’ motivational and affective factors mediate the relationship between stress and short video addiction. As indicated by the preceding path analysis, social interaction does not influence immersion. Therefore, perceived pressure does not affect short video addiction through a serial mediating pathway consisting of social interaction and immersion. Nevertheless, it is worth emphasizing that perceived stress can influence short video addiction through three distinct serial mediation pathways: the pathway involving social interaction and attitude, the pathway linking relaxing entertainment and attitude, and the pathway connecting relaxing entertainment and immersion. The identified pathways illustrate the complex mechanisms through which stress can indirectly lead to addictive behaviors, specifically in the context of short video consumption. When individuals experience stress, they may seek social interaction and relaxation through short video platforms as a coping mechanism (Sun et al., 2024). These social interactions and relaxing experiences can shape their attitudes toward such platforms, potentially fostering a more positive outlook that encourages sustained use (Curras-Perez et al., 2014). This positive attitude can, in turn, contribute to the development of addictive patterns of behavior. Similarly, individuals under stress may find short videos to be a source of relaxation and escapism (Vaterlaus and Winter, 2021). The immersive nature of these videos can draw users deeper into the content, enhancing their engagement and diminishing their awareness of the external environment (Miranda et al., 2023). This heightened level of immersion can facilitate the transition from casual use to addictive behavior, further exacerbating the cycle of stress and digital media consumption.

Lastly, the PLS-MGA results showed that there are significant differences between the three age groups on the effect of perceived stress on short video addiction and relaxing entertainment, attitude on short video addiction, and immersion on short video addiction. These findings underscore the role of age in shaping these relationships. Specifically, the effects of perceived stress on short video addiction were notable among users aged 21–25 and those older than 25, but were not significant for users aged 14–20. Moreover, the positive influence of immersion on short video addiction is more pronounced among users aged 14–20. One potential explanation is that younger users, especially adolescent users, have lower self-control and find it harder to disengage from watching short videos absorbedly, making them more susceptible to addiction (Lu et al., 2022; Li et al., 2021; Ma et al., 2020; Martins et al., 2020). This implies that individuals with diminished self-control capacities are more prone to becoming deeply immersed in digital environments, especially adolescents. Consistent with this finding, prior research has demonstrated that self-control negatively predicts immersion among adolescents (Ko et al., 2021). Self-control is typically conceptualized as an “individual’s motivation and capacity to inhibit or override a desire that stands in conflict with an endorsed self-regulatory goal or value” (Hofmann et al., 2017, p.5). Immersion reflects “a state of deep mental involvement” (Agrawal et al., 2020, p. 404). Furthermore, the effects of self-control and immersion in internet addiction manifest in different directions. Existing studies have illustrated that self-control is negatively related to digital addiction (Błachnio and Przepiorka, 2016; Li et al., 2021). In contrast, immersion has a positive impact on short video addiction (Tian et al., 2023; Yan et al., 2023). Besides, perceived stress has the most substantial effect on relaxation among users older than 25, indicating that managing stress and seeking relaxation are primary needs for this age group. Attitude toward short video content significantly affects addiction in younger users, but this effect is not significant for those aged over 25. The PLS-MGA results did not reveal significant differences between the age groups regarding the effects of perceived stress on social interaction, relaxing entertainment on attitude and immersion, and social interaction on immersion and attitude.

## 6 Implications

### 6.1 Theoretical implications

The empirical findings of the present study have two theoretical implications. First, this study introduces an E-CIU framework to elucidate users’ addiction toward short video applications. Previous research has predominantly focused on explaining addictive behavior using a motivational approach, neglecting a comprehensive investigation into affective path. In our framework, we integrate both psychological (i.e., stress), compensatory motivations (i.e., social interaction and relaxing entertainment), and affective responses (i.e., immersion and attitude) into an extended model to examine the impact paths of stress on addictive behavior. Consequently, this research establishes an innovative theoretical framework for understanding the underlying mechanisms in the relationship between stress and short video addiction. Secondly, we conducted a detailed analysis of the intricate interplay between these influencing factors among different groups. The results of this study contribute to the short video addiction literature by highlighting that stress has a great influence on short video addiction among different age groups.

### 6.2 Practical implications

This research also has two key practical implications. Firstly, the study highlights the role of stress perception in contributing to short video addiction. The findings reveal that stress directly influences users’ propensity for addiction to short videos. Stress affects users’ affective experiences, which in turn indirectly influence their immersion in short video content. Given that short videos have become a significant outlet for stress relief in modern life, it is crucial for designers of these applications to implement features that help users monitor and manage their viewing time. Providing prompts or tools to encourage users to take breaks could address their need for intermittent disconnection.

Secondly, targeted interventions should be developed for different user groups. For adolescents, strategies could include limiting their mobile and short video viewing time to prevent excessive use. For college students, educational programs that raise awareness about the risks and consequences of short video addiction could enhance self-awareness and self-control. For working professionals, promoting effective time management and encouraging alternative stress-relief activities, such as reading, exercising, or socializing, could help mitigate perceived stress and reduce reliance on short videos.

### 6.3 Limitations and further study

This study possesses certain limitations. Firstly, the quantitative methods employed in online surveys may not fully capture the comprehensive nature of users’ short video addiction reasons in China. Future studies could consider using interviews and grounded theory to obtain a more systematic and profound understanding of the underlying reasons for users’ addiction to short videos. Secondly, our sample predominantly comprised young individuals (aged 14–30), whereas China’s short video users represent a diverse demographic with varying addictive inclinations across different age groups. In the future, it would be advantageous to recruit a broader and more diverse range of participants from various age groups (i.e., Gen X; Gen Y; Gen Z). Finally, the present study adopted a cross-sectional survey design, which presents challenges in establishing causal relationships between variables. Future research efforts could use long-term longitudinal studies to investigate the impact of these variables over time.

## Data Availability

The raw data supporting the conclusions of this article will be made available by the authors, without undue reservation.

## Footnotes

^1^www.wjx.cn

## References

[ref1] Agrawal S. Simon A. Bech S. Bærentsen K. Forchhammer S. (2020). Defining immersion: literature review and implications for research on audiovisual experiences. J. Audio Eng. Soc. 68, 404–417. doi: 10.17743/jaes.2020.0039

[ref2] Aguinis H. Edwards J. R. Bradley K. J. (2017). Improving our understanding of moderation and mediation in strategic management research. Organ. Res. Methods 20, 665–685. doi: 10.1177/1094428115627498

[ref3] Ajzen I. (2002). Residual effects of past on later behavior: habituation and reasoned action perspectives. Personal. Soc. Psychol. Rev. 6, 107–122. doi: 10.1207/s15327957pspr0602_02

[ref4] Alloway T. P. Alloway R. G. (2012). The impact of engagement with social networking sites (SNSs) on cognitive skills. Comput. Hum. Behav. 28, 1748–1754. doi: 10.1016/j.chb.2012.04.015

[ref5] Bagozzi R. P. Yi Y. (1988). On the evaluation of structural equation models. J. Acad. Market Sci. 16, 74–94. doi: 10.1007/BF02723327

[ref6] Birditt K. S. Turkelson A. Fingerman K. L. Polenick C. A. Oya A. (2021). Age differences in stress, life changes, and social ties during the COVID-19 pandemic: implications for psychological well-being. Gerontologist 61, 205–216. doi: 10.1093/geront/gnaa204, PMID: 33346806 PMC7799124

[ref7] Bizer G. Y. Krosnick J. A. (2001). Exploring the structure of strength-related attitude features: the relation between attitude importance and attitude accessibility. J. Pers. Soc. Psychol. 81, 566–586. doi: 10.1037/0022-3514.81.4.566, PMID: 11642346

[ref8] Błachnio A. Przepiorka A. (2016). Dysfunction of self-regulation and self-control in Facebook addiction. Psychiat. Quart. 87, 493–500. doi: 10.1007/s11126-015-9403-1, PMID: 26589423 PMC4945680

[ref9] Brand M. Young K. S. Laier C. Wölfling K. Potenza M. N. (2016). Integrating psychological and neurobiological considerations regarding the development and maintenance of specific internet-use disorders: an interaction of person-affect-cognition-execution (I-PACE) model. Neurosci. Biobehav. Rev. 71, 252–266. doi: 10.1016/j.neubiorev.2016.08.033, PMID: 27590829

[ref10] Can L. Kaya N. (2016). Social networking sites addiction and the effect of attitude towards social network advertising. Proc. Soc. Behav. Sci. 235, 484–492. doi: 10.1016/j.sbspro.2016.11.059

[ref11] Cannito L. Annunzi E. Viganò C. Dell’Osso B. Vismara M. Sacco P. L.. (2022). The role of stress and cognitive absorption in predicting social network addiction. Brain Sci. 12:643. doi: 10.3390/brainsci12050643, PMID: 35625029 PMC9139642

[ref12] Caplan S. E. (2005). A social skill account of problematic internet use. J. Commun. 55, 721–736. doi: 10.1111/j.1460-2466.2005.tb03019.x

[ref13] Chak K. Leung L. (2004). Shyness and locus of control as predictors of internet addiction and internet use. Cyberpsychol. Behav. 7, 559–570. doi: 10.1089/cpb.2004.7.559 15667051

[ref14] Chatterjee S. (2021). Impact on addiction of online platforms on quality of life: age and gender as moderators. Aust. J. Inf. Syst. 25:2761. doi: 10.3127/ajis.v25i0.2761

[ref15] Cheah J. H. Thurasamy R. Memon M. A. Chuah F. Ting H. (2020). Multigroup analysis using SmartPLS: step-by-step guidelines for business research. Asian. J. Bus. Res. 10, 1–19. doi: 10.14707/ajbr.200087

[ref16] Chen C. C. Lin Y. C. (2018). What drives live-stream usage intention? The perspectives of flow, entertainment, social interaction, and endorsement. Telemat. Inform. 35, 293–303. doi: 10.1016/j.tele.2017.12.003

[ref17] Chen S. Weng L. Su Y. (2003). Development of Chinese internet addiction scale and its psychometric study. Chin. J. Psychol. 45, 279–294. doi: 10.1037/t44491-000

[ref18] Cheng K. H. Tsai C. C. (2020). Students’ motivational beliefs and strategies, perceived immersion and attitudes towards science learning with immersive virtual reality: a partial least squares analysis. Brit. J. Educ. Technol. 51, 2140–2159. doi: 10.1111/bjet.12956

[ref19] Chin W. W. (1998). Issues and opinion on structural equation modeling. MIS. Quart. 22, 7–17. doi: 10.5555/290231.290235

[ref20] China Internet Network Information Center. (2023). The 53rd China Internet Network Information Center Statistical Report on Internet Development. Available at: https://www.cnnic.net.cn/NMediaFile/2024/0325/MAIN1711355296414FIQ9XKZV63.pdf (Accessed March 22, 2024).

[ref21] Cohen S. Kamarch T. Mermelstein R. (1983). A global measure of perceived stress. J. Health Soc. Behav. 24:385e396. doi: 10.2307/2136404 6668417

[ref22] Cohen S. Kessler R. C. Gordon L. U. (1995). “Strategies for measuring stress in studies of psychiatric and physical disorders” in Measuring stress: A guide for health and social scientist. eds. Cohen S. Kessler R. C. Gordon L. U. (New York: Oxford University Press), 3–26.

[ref23] Csikszentmihalyi M. (1990). Flow: The psychology of optimal experience. New York: Harper Perennial.

[ref24] Cui Y. Zhu J. Liu Y. (2022). Exploring the social and systemic influencing factors of mobile short video applications on the consumer urge to buy impulsively. J. Glob. Inf. Manag. 30, 1–23. doi: 10.4018/JGIM.301201

[ref25] Curras-Perez R. Ruiz-Mafe C. Sanz-Blas S. (2014). Determinants of user behaviour and recommendation in social networks. Ind. Manage. Data. Syst. 114, 1477–1498. doi: 10.1108/imds-07-2014-0219

[ref26] Da-Yong Z. Zhan S. (2022). Short video users’ personality traits and social sharing motivation. Front. Psychol. 13:1046735. doi: 10.3389/fpsyg.2022.1046735, PMID: 36571026 PMC9784466

[ref27] Deng T. Vargas-Bianchi L. Mensa M. (2023). Cross-cultural comparison of TikTok uses and gratifications. Behav. Inform. Technol. 43, 3047–3059. doi: 10.1080/0144929X.2023.2270534

[ref28] Devine D. Ogletree A. M. Shah P. Katz B. (2022). Internet addiction, cognitive, and dispositional factors among US adults. Comput. Human Behav. Rep. 6:100180. doi: 10.1016/j.chbr.2022.100180

[ref29] Dholakia U. M. Bagozzi R. P. Pearo L. K. (2004). A social influence model of consumer participation in network-and small-group-based virtual communities. Int. J. Res. Mark. 21, 241–263. doi: 10.1016/j.ijresmar.2003.12.004

[ref30] Dong X. Wen X. Chang Y. Li H. (2024). How do short video content characteristics influence short video app addiction? An affective response perspective. Int. J. Mob. Commun. 23, 425–449. doi: 10.1504/IJMC.2024.138782

[ref31] Dong Z. Xie T. (2024). Why do people love short-form videos? The motivations for using Chinese TikTok (Douyin) and implications for well-being. Curr. Psychol. 43, 22283–22296. doi: 10.1007/s12144-024-05927-4

[ref32] Eagly A. H. Chaiken S. (1993). The psychology of attitudes. San Diego: Harcourt Brace Jovanovich College Publishers.

[ref33] Edwards K. (1990). The interplay of affect and cognition in attitude formation and change. J. Pers. Soc. Psychol. 59, 202–216. doi: 10.1037/0022-3514.59.2.202

[ref34] Fornell C. Larcker D. F. (1981). Evaluating structural equation models with unobservable variables and measurement error. J. Mark. Res. 18, 39–50. doi: 10.1177/002224378101800104

[ref35] Geisser S. (1975). The predictive sample reuse method with applications. J. Am. Stat. Assoc. 70, 320–328. doi: 10.1080/01621459.1975.10479865

[ref36] Gong Z. Wang L. Wang H. (2021). Perceived stress and internet addiction among Chinese college students: mediating effect of procrastination and moderating effect of flow. Front. Psychol. 12:632461. doi: 10.3389/fpsyg.2021.632461, PMID: 34262501 PMC8273309

[ref37] Hair J. F. Matthews L. M. Matthews R. L. Sarstedt M. (2017). PLS-SEM or CB-SEM: updated guidelines on which method to use. Int. J. Multivariate Data Anal. 1, 107–123. doi: 10.1504/IJMDA.2017.087624

[ref38] Hamari J. Shernoff D. J. Rowe E. Coller B. Asbell-Clarke J. Edwards T. (2016). Challenging games help students learn: an empirical study on engagement, flow and immersion in game-based learning. Comput. Hum. Behav. 54, 170–179. doi: 10.1016/j.chb.2015.07.045

[ref39] Henseler J. Hubona G. Ray P. A. (2016). Using PLS path modeling in new technology research: updated guidelines. Ind. Manag. Data Syst. 116, 2–20. doi: 10.1108/IMDS-09-2015-0382

[ref40] Henseler J. Ringle C. M. Sarstedt M. (2015). A new criterion for assessing discriminant validity in variance-based structural equation modeling. J. Acad. Market Sci. 43, 115–135. doi: 10.1007/s11747-014-0403-8

[ref41] Henter R. (2014). Affective factors involved in learning a foreign language. Proc. Soc. Behav. Sci. 127, 373–378. doi: 10.1016/j.sbspro.2014.03.274

[ref42] Heo J. Oh J. Subramanian S. V. Kim Y. Kawachi I. (2014). Addictive internet use among Korean adolescents: a national survey. PLoS One 9:e87819. doi: 10.1371/journal.pone.0087819, PMID: 24505318 PMC3914839

[ref43] Hofmann W. Reinecke L. Meier A. (2017). “Of sweet temptations and bitter aftertaste: self-control as a moderator of the effects of media use on well-being” in The Routledge handbook of media use and well-being: International perspectives on theory and research on positive media effects. eds. Reinecke L. Oliver M. B. (London: Routledge), 211–222.

[ref44] Howarth C. (2006). How social representations of attitudes have informed attitude theories: the consensual and the reified. Theor. Psychol. 16, 691–714. doi: 10.1177/0959354306067443

[ref45] Huang Q. Hu M. Chen H. (2021). Exploring stress and problematic use of short-form video applications among middle-aged Chinese adults: the mediating roles of duration of use and flow experience. Int. J. Env. Res. Pub. He. 19:132. doi: 10.3390/ijerph19010132, PMID: 35010389 PMC8751076

[ref46] Ioannidis K. Treder M. S. Chamberlain S. R. Kiraly F. Redden S. A. Stein D. J.. (2018). Problematic internet use as an age-related multifaceted problem: evidence from a two-site survey. Addict. Behav. 81, 157–166. doi: 10.1016/j.addbeh.2018.02.017, PMID: 29459201 PMC5849299

[ref47] Jennett C. Cox A. L. Cairns P. Dhoparee S. Epps A. Tijs T.. (2008). Measuring and defining the experience of immersion in games. Int. J. Hum. Comput. St. 66, 641–661. doi: 10.1016/j.ijhcs.2008.04.004

[ref48] Jeong E. J. Kim D. H. (2011). Social activities, self-efficacy, game attitudes, and game addiction. Cyberpsychol. Behav. Soc. Netw. 14, 213–221. doi: 10.1089/cyber.2009.0289, PMID: 21067285

[ref49] Jiang L. Yoo Y. (2024). Adolescents’ short-form video addiction and sleep quality: the mediating role of social anxiety. BMC Psychol. 12:369. doi: 10.1186/s40359-024-01865-9, PMID: 38943173 PMC11214215

[ref50] Karagiannidis Y. Barkoukis V. Gourgoulis V. Kosta G. Antoniou P. (2015). The role of motivation and metacognition on the development of cognitive and affective responses in physical education lessons: a self-determination approach. Motricidade 11, 135–150. doi: 10.6063/motricidade.3661

[ref51] Kardefelt-Winther D. (2014a). A conceptual and methodological critique of internet addiction research: towards a model of compensatory internet use. Comput. Hum. Behav. 31, 351–354. doi: 10.1016/j.chb.2013.10.059

[ref52] Kardefelt-Winther D. (2014b). The moderating role of psychosocial well-being on the relationship between escapism and excessive online gaming. Comput. Hum. Behav. 38, 68–74. doi: 10.1016/j.chb.2014.05.020

[ref53] Khan M. L. (2017). Social media engagement: what motivates user participation and consumption on YouTube? Comput. Hum. Behav. 66, 236–247. doi: 10.1016/j.chb.2016.09.024

[ref54] Kim J. Hong H. Lee J. Hyun M. H. (2017). Effects of time perspective and self-control on procrastination and internet addiction. J. Behav. Addict. 6, 229–236. doi: 10.1556/2006.6.2017.017, PMID: 28494615 PMC5520116

[ref55] Ko J. U. Kim K. H. Jeon J. H. (2021). The effects of self-control on internet immersion: the moderating effects of parenting attitude. Asia Pac. J. Res. Interchange 7, 47–57. doi: 10.47116/apjcri.2021.09.05

[ref9001] Lai I. H. Kim D. J. Jeong E. J. (2016). Online digital game addiction: How does social relationship impact game addiction?. in AMCIS 2016: Surfing the IT innovation wave - 22nd Americas conference on information systems (San Diego, CA), 1–18.

[ref56] LaRose R. Lin C. A. Eastin M. S. (2003). Unregulated internet usage: addiction, habit, or deficient self-regulation? Media Psychol. 5, 225–253. doi: 10.1207/S1532785XMEP0503_01

[ref57] Lazarus R. S. (1993). From psychological stress to the emotions: a history of changing outlooks. Annu. Rev. Psychol. 44, 1–22. doi: 10.1146/annurev.ps.44.020193.000245 8434890

[ref58] Lee Z. W. Cheung C. M. Chan T. K. (2021). Understanding massively multiplayer online role-playing game addiction: a hedonic management perspective. Inform. Syst. J. 31, 33–61. doi: 10.1111/isj.12292

[ref59] Lee H. M. Li B. J. (2023). So far yet so near: exploring the effects of immersion, presence, and psychological distance on empathy and prosocial behavior. Int. J. Hum. Comput. St. 176:103042. doi: 10.1016/j.ijhcs.2023.103042

[ref60] Lehenbauer-Baum M. Klaps A. Kovacovsky Z. Witzmann K. Zahlbruckner R. Stetina B. U. (2015). Addiction and engagement: an explorative study toward classification criteria for internet gaming disorder. Cyberpsych. Beh. Soc. N. 18, 343–349. doi: 10.1089/cyber.2015.0063, PMID: 26075922

[ref61] Li S. Ren P. Chiu M. M. Wang C. Lei H. (2021). The relationship between self-control and internet addiction among students: a meta-analysis. Front. Psychol. 12:735755. doi: 10.3389/fpsyg.2021.735755, PMID: 34899477 PMC8653951

[ref62] Lin Y. Wang G. Suh A. (2020). “Exploring the effects of immersive virtual reality on learning outcomes: a two-path model” in Augmented cognition. Human cognition and behavior. eds. Schmorrow D. Fidopiastis C. (Berlin: Springer International Publishing), 86–105.

[ref63] Liu C. C. Chang I. C. (2016). Model of online game addiction: the role of computer-mediated communication motives. Telemat. Inform. 33, 904–915. doi: 10.1016/j.tele.2016.02.002

[ref64] Liu Y. Ni X. Niu G. (2021). Perceived stress and short-form video application addiction: a moderated mediation model. Front. Psychol. 12:747656. doi: 10.3389/fpsyg.2021.747656, PMID: 35002843 PMC8733249

[ref65] Lu L. Liu M. Ge B. Bai Z. Liu Z. (2022). Adolescent addiction to short video applications in the mobile internet era. Front. Psychol. 13:893599. doi: 10.3389/fpsyg.2022.893599, PMID: 35619797 PMC9127662

[ref66] Luchman J. N. Bergstrom J. Krulikowski C. (2014). A motives framework of social media website use: a survey of young Americans. Comput. Hum. Behav. 38, 136–141. doi: 10.1016/j.chb.2014.05.016

[ref67] Lv X. Zhang R. Su Y. Yang Y. (2022). Exploring how live streaming affects immediate buying behavior and continuous watching intention: a multigroup analysis. J. Travel Tour. Mark. 39, 109–135. doi: 10.1080/10548408.2022.2052227

[ref68] Ma S. Huang Y. Ma Y. (2020). Childhood maltreatment and mobile phone addiction among Chinese adolescents: loneliness as a mediator and self-control as a moderator. Front. Psychol. 11:813. doi: 10.3389/fpsyg.2020.00813, PMID: 32477211 PMC7235189

[ref69] Marcinkowski T. Reid A. (2019). Reviews of research on the attitude-behavior relationship and their implications for future environmental education research. Environ. Educ. Res. 25, 459–471. doi: 10.1080/13504622.2019.1634237

[ref70] Martins M. V. Formiga A. Santos C. Sousa D. Resende C. Campos R.. (2020). Adolescent internet addiction-role of parental control and adolescent behaviours. Int. J. Pediatr. Adolesc. Med. 7, 116–120. doi: 10.1016/j.ijpam.2019.12.003 33094139 PMC7568070

[ref71] Matthews L. (2017). “Applying multigroup analysis in PLS-SEM: a step-by-step process” in Partial least squares path modeling. eds. Latan H. Noonan R. (Berlin: Springer), 219–243.

[ref72] McNally J. Harrington B. (2017). How millennials and teens consume mobile video. In proceedings of the 2017 ACM international conference on interactive experiences for TV and online video, 31–39.

[ref73] Miranda S. Trigo I. Rodrigues R. Duarte M. (2023). Addiction to social networking sites: motivations, flow, and sense of belonging at the root of addiction. Technol. Forecast. Soc. 188:122280. doi: 10.1016/j.techfore.2022.122280

[ref74] Mouchabac S. Maatoug R. Conejero I. Adrien V. Bonnot O. Millet B.. (2021). In search of digital dopamine: how apps can motivate depressed patients, a review and conceptual analysis. Brain Sci. Nov. 11:1454. doi: 10.3390/brainsci11111454, PMID: 34827453 PMC8615613

[ref75] Mu H. Jiang Q. Xu J. Chen S. (2022). Drivers and consequences of short-form video (SFV) addiction amongst adolescents in China: stress-coping theory perspective. Int. J. Environ. Res. Public Health 19:14173. doi: 10.3390/ijerph192114173, PMID: 36361050 PMC9658094

[ref76] National Radio and Television Administration. (2024). China Network Audio-visual Development Research Report. Available at: https://news.cnr.cn/native/gd/20240328/t20240328_526643305.shtml (Accessed March 27, 2024).

[ref77] Nong W. He Z. Ye J. H. Wu Y. F. Wu Y. T. Ye J. N.. (2023). The relationship between short video flow, addiction, serendipity, and achievement motivation among Chinese vocational school students: the post-epidemic era context. Healthcare 11:462. doi: 10.3390/healthcare11040462, PMID: 36832995 PMC9957412

[ref78] Ozel M. Caglak S. Erdogan M. (2013). Are affective factors a good predictor of science achievement? Examining the role of affective factors based on PISA 2006. Learn. Individ. Differ. 24, 73–82. doi: 10.1016/j.lindif.2012.09.006

[ref79] Pang H. (2021). Identifying associations between mobile social media users’ perceived values, attitude, satisfaction, and eWOM engagement: the moderating role of affective factors. Telemat. Inform. 59:101561. doi: 10.1016/j.tele.2020.101561

[ref80] Pavić J. Rijavec M. (2013). Stress and television viewing in female college students: mediating role of TV viewing motives and TV affinity. Suvremena Psihologija 16, 33–47.

[ref81] Petty R. E. Briñol P. (2015). Emotion and persuasion: cognitive and meta-cognitive processes impact attitudes. Cogn. Emot. 29, 1–26. doi: 10.1080/02699931.2014.967183, PMID: 25302943

[ref83] Qin Y. Omar B. Musetti A. (2022). The addiction behavior of short-form video app TikTok: the information quality and system quality perspective. Front. Psychol. 13:932805. doi: 10.3389/fpsyg.2022.932805, PMID: 36148123 PMC9486470

[ref84] Qu D. Liu B. Jia L. Zhang X. Chen D. Zhang Q.. (2024). The longitudinal relationships between short video addiction and depressive symptoms: a cross-lagged panel network analysis. Comput. Hum. Behav. 152:108059. doi: 10.1016/j.chb.2023.108059

[ref85] Rémond J. Romo L. (2019). Analysis of gambling in the media related to screens: immersion as a predictor of excessive use? Int. J. Environ. Res. Public Health 15:58. doi: 10.3390/ijerph15010058, PMID: 29301311 PMC5800157

[ref86] Saneinia S. Zhou R. Gholizadeh A. Asmi F. (2022). Immersive media-based tourism emerging challenge of VR addiction among generation Z. Front. Public Health 10:833658. doi: 10.3389/fpubh.2022.833658, PMID: 35844881 PMC9286390

[ref87] Schiffrin H. H. Nelson S. K. (2008). Stressed and happy? Investigating the relationship between happiness and perceived stress. J. Happiness Stud. 11, 33–39. doi: 10.1007/s10902-008-9104-7

[ref88] Seah M. L. Cairns P. (2008). “From immersion to addiction in videogames,” in Proceedings of the 22nd British HCI group annual conference on people and computers: Culture, creativity, interaction, 55–63.

[ref89] Shen C. Williams D. (2011). Unpacking time online: connecting internet and massively multiplayer online game use with psychosocial well-being. Commun. Res. 38, 123–149. doi: 10.1177/0093650210377196

[ref90] Shi J. Ali M. Chew F. (2024). Understanding gratifications for engaging with short-video: a comparison of TikTok use in the USA and China. Int. J. Mob. Commun. 23, 175–200. doi: 10.1504/IJMC.2024.136627

[ref91] Stanković M. Nešić M. Čičević S. Shi Z. (2021). Association of smartphone use with depression, anxiety, stress, sleep quality, and internet addiction. Empirical evidence from a smartphone application. Pers. Individ. Dif. 168:110342. doi: 10.1016/j.paid.2020.110342

[ref93] Sun R. Zhang M. X. Yeh C. Ung C. O. L. Wu A. M. (2024). The metacognitive-motivational links between stress and short-form video addiction. Technol. Soc. 77:102548. doi: 10.1016/j.techsoc.2024.102548

[ref95] Tian X. Bi X. Chen H. (2023). How short-form video features influence addiction behavior? Empirical research from the opponent process theory perspective. Inform. Technol. Peopl. 36, 387–408. doi: 10.1108/itp-04-2020-0186

[ref96] Toh W. X. Ng W. Q. Yang H. Yang S. (2023). Disentangling the effects of smartphone screen time, checking frequency, and problematic use on executive function: a structural equation modelling analysis. Curr. Psychol. 42, 4225–4242. doi: 10.1007/s12144-021-01759-8

[ref97] Tsai C. C. Lin S. S. (2001). Analysis of attitudes toward computer networks and internet addiction of Taiwanese adolescents. Cyberpsychol. Behav. 4, 373–376. doi: 10.1089/109493101300210277, PMID: 11710262

[ref98] Vaterlaus J. M. Winter M. (2021). TikTok: an exploratory study of young adults’ uses and gratifications. Soc. Sci. J. 9, 1–20. doi: 10.1080/03623319.2021.1969882

[ref99] Velezmoro R. Lacefield K. Roberti J. (2010). Perceived stress, sensation seeking, and college students’ abuse of the internet. Comput. Hum. Behav. 26, 1526–1530. doi: 10.1016/j.chb.2010.05.020

[ref102] Yan Y. He Y. Li L. (2023). Why time flies? The role of immersion in short video usage behavior. Front. Psychol. 14:1127210. doi: 10.3389/fpsyg.2023.1127210, PMID: 37123290 PMC10140313

[ref103] Yang Z. Griffiths M. D. Yan Z. Xu W. (2021). Can watching online videos be addictive? A qualitative exploration of online video watching among Chinese young adults. Int. J. Environ. Res. Public Health 18:7247. doi: 10.3390/ijerph18147247, PMID: 34299696 PMC8306552

[ref104] Ye J. H. He Z. Yang X. Lee Y. S. Nong W. Ye J. N.. (2023). Predicting the learning avoidance motivation, learning commitment, and silent classroom behavior of Chinese vocational college students caused by short video addiction. Healthcare 11:985. doi: 10.3390/healthcare11070985, PMID: 37046912 PMC10094292

[ref105] Zadeh A. H. Farhang M. Zolfagharian M. Hofacker C. F. (2023). Predicting value cocreation behavior in social media via integrating uses and gratifications paradigm and theory of planned behavior. J. Res. Interact. Mark. 17, 195–214. doi: 10.1108/JRIM-10-2020-0209

[ref106] Zhang P. (2013). The affective response model: a theoretical framework of affective concepts and their relationships in the ICT context. MIS. Quart. 37, 247–274. doi: 10.25300/MISQ/2013/37.1.11

[ref107] Zhang N. Hazarika B. Chen K. Shi Y. (2023). A cross-national study on the excessive use of short-video applications among college students. Comput. Hum. Behav. 145:107752. doi: 10.1016/j.chb.2023.107752

[ref108] Zhang X. Wu Y. Liu S. (2019). Exploring short-form video application addiction: socio-technical and attachment perspectives. Telemat. Inform. 42:101243. doi: 10.1016/j.tele.2019.101243

